# Research on Friction Performance of Friction Stir Welding Tools Based on Non-Smooth Structure

**DOI:** 10.3390/biomimetics9070427

**Published:** 2024-07-13

**Authors:** Yupeng Li, Yu Huangfu, Jiacheng Feng, Limei Tian, Luquan Ren

**Affiliations:** 1 Key Laboratory of Bionic Engineering, Ministry of Education, Jilin University, Changchun 130025, China; lmtian@jlu.edu.cn (L.T.); lqren@jlu.edu.cn (L.R.); 2 Key Laboratory of Advanced Structural Materials, Ministry of Education, Changchun University of Technology, Changchun 130012, China; hfyfighting123@163.com (Y.H.); fengjc1208@163.com (J.F.); 3 College of Materials Science and Engineering, Changchun University of Technology, Changchun 130012, China; 4 College of Biological and Agricultural Engineering, Jilin University, Changchun 130025, China

**Keywords:** non-smooth structure, friction stir welding, tool, friction characteristics

## Abstract

In this study, based on the principles of bionics, we fabricated a bionic non-smooth concave pit structure on the shoulders of friction stir welding tools and detected the thermal cycling curve, downforce, and torque of the tool in the welding process. We tested the wear loss weight and analyzed the surface morphology of the shoulder surfaces after welding for 200 m. This study found that as the distance between the concave pits decreased and the number of concave pits increased, the maximum downforce, torque, and temperature in the welding process showed a decreasing trend. As the speed increased, no matter how the tool structure changed, the downforce and torque decreased, while the peak thermal cycle temperature increased. The experimental welding results show that the wear loss weight of the non-smooth structure tool significantly reduced. The lowest wear loss weight of the tool with a concave pit interval of 1.125 mm was only 0.1529 g, which is 27% lower than that of the conventional tool. Our observations of the surface morphology of the tool shoulder after welding showed that the amount of aluminum swarf on the tool shoulder of the welding tool gradually declined with the increasing density of the uneven pits. The lowest number of aluminum chips adhered to a welding tool with a pit distance of 1.125 mm. Therefore, friction stir welding tools with biomimetic structures have better wear resistance and adhesion resistance.

## 1. Introduction

Friction stir welding (FSW) is a solid-state welding technology [1,2,3] that has been widely applied in various fields due to its characteristics of creating no metallurgical defects, fast welding speed, and high degree of automation. The welding tool, as the core component of FSW, plays a crucial role in the performance of the joint [4]. The current research on tools mainly focuses on improving joint performance through the design and optimization of profiles. Kumar [5] conducted a study on the effects of three different tool pin profiles (square, threaded cylinder, and straight cylinder) on the microstructure and mechanical strength of friction stir welded AA5083 and AA5754 alloy joints. The results indicated that the square tool exhibited more favorable characteristics. Azmi’s research [6] suggested that the joints produced with the threaded tapered cylindrical pin profile showed the highest tensile strength and better mixing characteristics based on its hardness distribution compared to the joints produced using threaded straight cylindrical and tapered cylindrical pin profiles. Dawood’s research [7] found that using different tool pin profiles had significant effects on weld joints, with the triangular tool pin profile demonstrating superior metallurgical and mechanical weld properties compared to other tool pin profiles. Mehta [8] analyzed the adhesion levels of various pin geometries and welding conditions based on machining theory while evaluating effective stresses on polygonal pins following the principles of mechanics. However, there has been little attention paid to how to improve the friction, wear performance [9,10,11] and service life of tools.

Non-smooth surfaces [12] are a type of body surface morphology with unique shapes found in nature. Current research on biological non-smooth surfaces indicates that they possess characteristics such as wear resistance and drag reduction [13,14,15]. The application of bionic technology [16,17] to incorporate non-smooth surfaces into production and daily life has yielded positive results, as seen in well-known examples like shark skin swimsuits and golf balls [18,19] within the field of fluid dynamics. In recent years, extensive research has been conducted by scholars to enhance the friction performance of bionic non-smooth microstructures [20,21]. Gu [22] suggests that a circular non-smooth surface structure with d = 1 mm can improve the cavitation distribution on blades, effectively reducing the amount of cavitation damage in centrifugal pumps and decreasing the area affected. Cheng [23] utilized a non-smooth concave surface structure to develop moisture management knitted fabrics, demonstrating that an appropriate unit size could improve moisture management properties while reducing fabric weight and thickness; however, mesh structures could enhance air permeability but weaken moisture management properties. Ma [24] applied two types of non-smooth structures to traditional screw conveyor blades, finding that the concave bionic non-smooth structure altered the particle flow state and improved the grain-conveying performance. Wang [25] showed that arranging non-smooth surfaces on the front and side surface of train tails slowed down the vortex motion at the tail end, shortened the wake influence regions, decreased the velocity fluctuation amplitude in wake regions, and reduced aerodynamic drag.

In conclusion, bionic non-smooth surfaces exhibit excellent friction and wear properties with a wide range of potential applications. However, there is currently a lack of relevant research in the area of friction stir welding. To address the practical requirements of wear resistance and drag reduction in welding tools, this study, building on the research from the aforementioned references [22,23,24,25], created a non-smooth concave pit structure on the friction stir welding tools. The thermal cycle curve during welding processes was studied along with the stress situations created during the joint wear resistance analysis—providing an innovative option for designing high-performance tools.

## 2. Materials and Methods

### 2.1. Materials

FSW tools used in the experiment were fabricated from H13 hot-work die steel with the chemical composition detailed in Table 1. The base metal (BM) used in this study was 6061-T6 aluminum alloy with a thickness of 4 mm in plate form. The chemical composition of the BM is presented in Table 2.

### 2.2. Bionic Structural Design

Numerous studies have demonstrated that non-smooth surface structures such as pits exhibit exceptional drag reduction and anti-sticking properties in natural organisms [19,22,24,26,27]. Considering the integrity, applicability, and practicality of selecting bionic non-smooth structures, along with the working characteristics and mechanical processing of the welding tool, this study opted to utilize concave non-smooth surfaces as the bionic feature.

**Figure 1:**
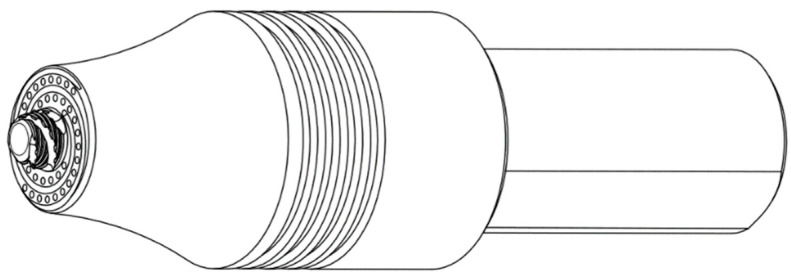
Design diagram of the welding tools based on bionic non-smooth structure.

**Figure 2:**
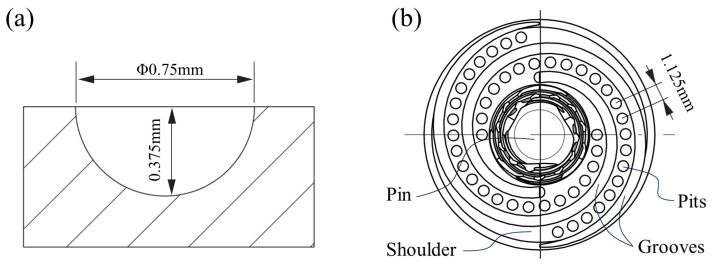
Design diagram of non-smooth structure, (**a**) pit size, (**b**) spiral grooves and pits distribution.

A design diagram of the experimental welding tools is shown in Figure 1, with a shoulder diameter of 16 mm and a pin length of 3.9 mm. Double Archimedean spiral grooves, 0.75 mm wide and 0.375 mm deep, were machined on the shoulder of the tools to maintain effective drive on high-temperature plastic metals during the welding process. Hemispherical pits were also machined in the plane between these spiral grooves to create a non-smooth surface with pit diameters and depths both of 0.75 mm (as shown in Figure 2a), while pit spacing was set at 3 mm, 1.5 mm, and 1.125 mm, respectively (as shown in Figure 2b). The root and end diameters of the pin were 7 mm and 5 mm, respectively, and the outer circle was machined with M6 × l mm threading.

**Figure 3:**
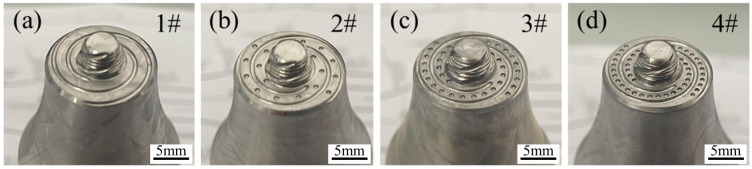
Experimental welding tools: (**a**) no pits, (**b**) pit spacing of 3 mm, (**c**) pit spacing of 1.5 mm, (**d**) pit spacing of 1.125 mm.

The prepared tools were labeled as 2#, 3#, and 4# based on center distances of 3 mm, 1.5 mm, and 1.125 mm for the pits, respectively. A tool with no pits in its structure was used as the control, labeled as 1# (as illustrated in Figure 3).

### 2.3. Experimental Equipment and Methods

**Figure 4:**
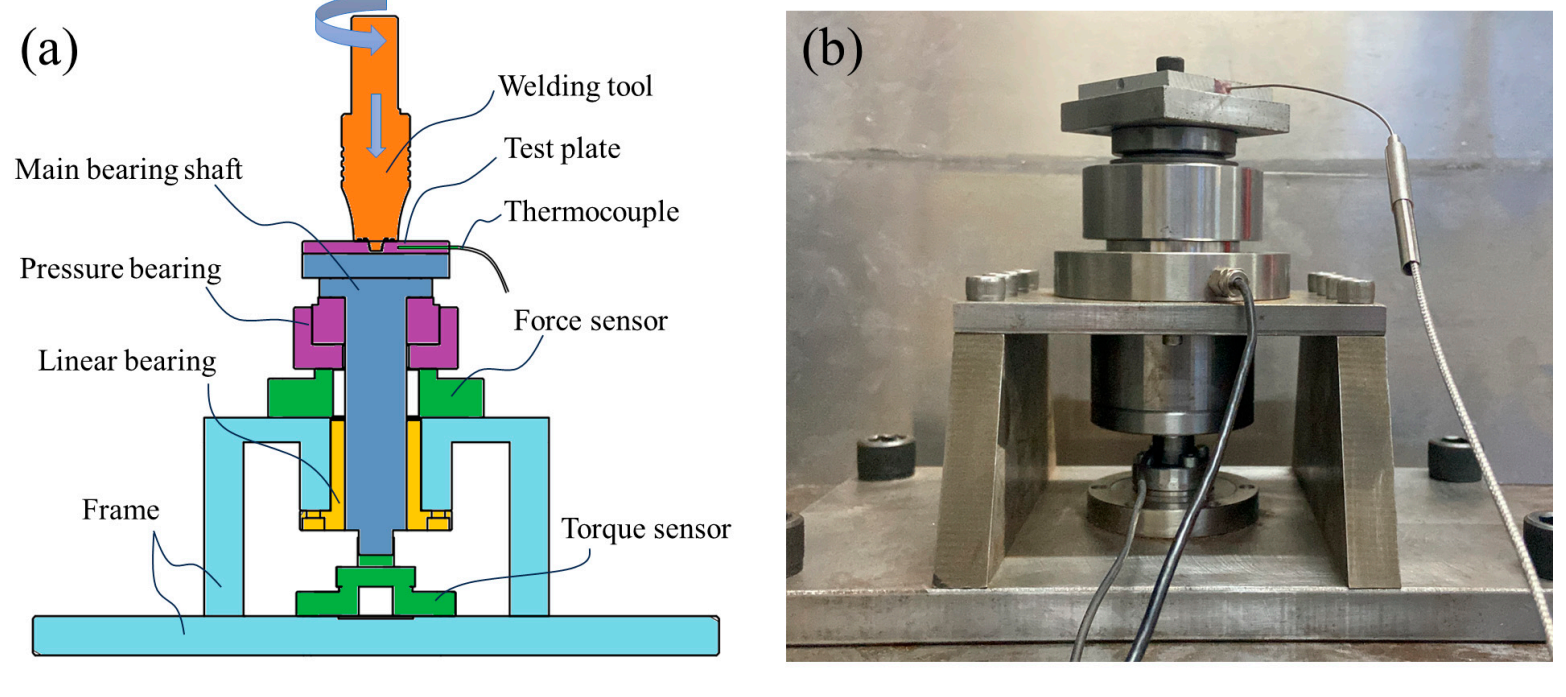
Comprehensive testing platform: (**a**) schematic diagram, (**b**) physical photos.

The friction stir welding (FSW) equipment model utilized in this experimental investigation was SCB-LM-3324-2D-13T. A self-designed comprehensive testing platform (as depicted in Figure 4) was employed to capture real-time thermal cycle curves, downforce, and torque during welding with non-smooth structure welding tools. The platform incorporated torque sensors, pressure sensors, K-type armored temperature sensors, fixed brackets, and multiple static strain gauges. During operation, the experimental plate was secured at the top of the platform while the temperature sensor was positioned 8 mm away from the center point of the welding spot and 2 mm from the surface of the plate.

When spot welding was used to evaluate the temperature and force of the welding tools, the rotation speed of the welding tools was set at 1000 r/min or 1500 r/min. The wear test was carried out at a speed of 1500 r/min and a welding speed of 500 mm/min. We conducted a 200 m practical welding operation with all welding tools. Every 50 m of welding, we removed aluminum debris from the tool surface and measured its weight using an FA2004N electronic balance, which has a minimum reading of 0.0001 g and a repeat accuracy of 0.0001 g. For each tool, we calculated the average of three measurements. Wear marks on the shoulder of the welding tools after welding were observed using a JSM-IT500 scanning electron microscope (SEM).

## 3. Results

### 3.1. Thermal Cycling and Stress Analysis during Welding Process

**Figure 5:**
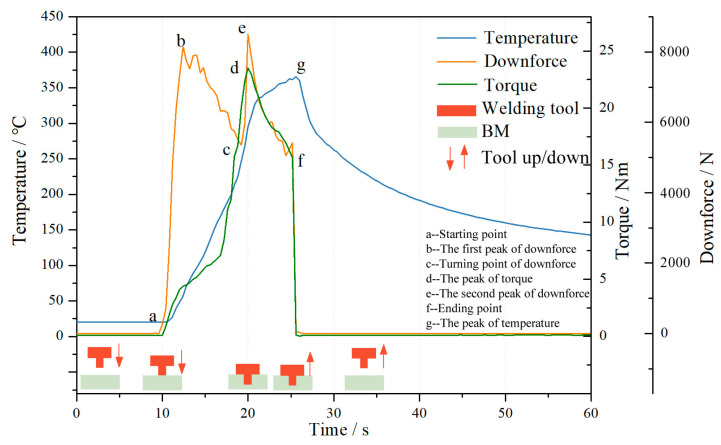
The temperature, torque, and downforce distribution of non-smooth structure during welding.

Figure 5 illustrates the distribution of temperature, torque, and downforce for a non-smooth structure 4# tool during fixed-point welding at 1000 r/min. According to the graph, it is evident that at the onset of welding, driven by the welding equipment, the welding tool rotates at a high speed and descends uniformly at a rate of 0.5 mm/s. Upon initial contact between the pin and the base material (point a), there is a rapid increase in downforce. As the pin penetrates to a depth of approximately 2 mm, the downforce reaches its first peak (point b) at 8148 N. Subsequently, due to continuous friction and stirring between the pin and base material, there is a gradual increase in temperature at the welding position of the base material. This leads to a decrease in strength with increasing temperature, resulting in a decline in downforce from its first peak to reach valley point c at 5355 N. Concurrently, during this process, the torque initially gradually increases before slowing down and then increasing again. At this stage of welding, as penetration continues, the temperature rises within the weld area causing the strength of the base material to decrease; torque also experiences an initial significant increase followed by a reduced rate of increase.

When the pin is about to fully penetrate the base material, the downforce is at its minimum. As the welding process continues, the shoulder of the tool begins to make contact with the base material and is pressed 0.5 mm below the surface. At this stage, there is a significant increase in the downward pressure, which reaches its maximum value of 8494.8 N during the welding process. The area of the shoulder is greater, and the torque generated by the contact with the base material is also increased. Therefore, the torque also increases rapidly at this point, reaching a maximum of 23.49 N·m when the shoulder is fully in contact with the base material. The temperature at the measurement point 8 mm from the center of the weld also increased rapidly, reaching 327.5 °C.

The welding program is designed to apply a downforce of 0.5 mm to press the shoulder onto the surface of the base material, rotate it at a fixed point for 5 s, and then gradually lift it off the base material. As a result, the continuous increase in temperature and the decrease in the strength of the base material, together with the high-speed rotation of the pin and shoulder, result in a decreasing trend in both downforce and torque once their maximum values are reached, even though the rotational speed is maintained. As the friction between the welding tool and the base material continues, heat accumulates, causing the temperature of the base material to rise continuously, albeit at a slower rate.

At the end of the fixed-point rotation, the welding tool will lift and detach from the base material at a speed of 2.5 mm/min. As the pin has a V-shaped design, when the tool detaches from the base material, both downforce and torque will rapidly decrease to 0 at point f (Figure 5). At this point the welding stops, and the heat generated by friction at the tool interface also stops immediately. The temperature at the 8 mm measurement point reaches its peak value of 365.4 °C and the temperatures in the central welding area begin to dissipate into the surrounding areas. The heat-sealing process then enters a cooling phase, causing the temperature to gradually decrease from the highest point on the curve.

### 3.2. Thermal Cycling and Force Analysis of Different Bionic Structures

**Figure 6:**
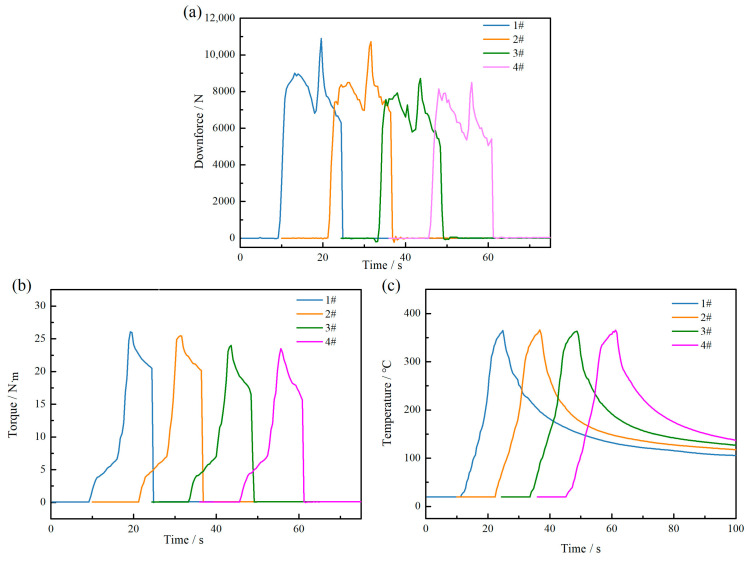
Comparison of downforce, torque, and temperature of different tools at 1000 r/min: (**a**) downforce; (**b**) torque; (**c**) temperature.

A comparison of the temperature, downforce, and torque of all tools during fixed-point welding at a rotation speed of 1000 r/min is shown in Figure 6. The graph illustrates that the changes in downforce, torque, and temperature of all tools exhibit similar curve shapes during the welding process, indicating minimal variation in the temperature and force fields. However, upon comparing the maximum values of all curves, it is evident that during the welding process with tool 1#, the peak downforce is 10,896 N, the peak torque is 26.09 N·m, and the peak temperature is 365 °C. In contrast to this, for tool 4#, the peak values are recorded as 8494.8 N for downward pressure, 23.5 N·m for torque, and 363.4 °C for temperature, respectively, showing significant changes. The temperature distribution of the four types of tools ranges from 366 °C to 362.7 °C, showing minimal variation. This indicates that the implementation of non-smooth pit structures on the shoulder of the welding tool can attenuate downforce, diminish friction, and reduce torque.

### 3.3. Thermal Cycling and Force Analysis of Different Rotational Speed

**Figure 7:**
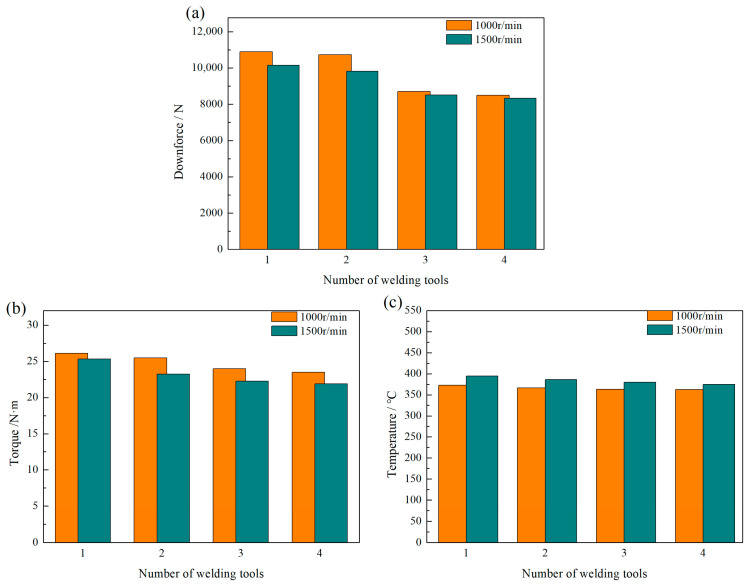
Comparison of peak values of downforce, torque, and temperature of different tools: (**a**) downforce; (**b**) torque; (**c**) temperature.

Based on the above research, the welding operation was carried out at a spindle speed of 1500 r/min. Downforce, torque, and temperature were measured during the welding process and compared with the peak values at 1000 r/min, as shown in Figure 7 below. The graph shows that the changes in downforce, torque, and temperature for different tools at 1500 r/min are similar to those at 1000 r/min. Smaller distances between the uneven pits result in lower peak values for downforce and torque and slightly lower peak values for temperature. When comparing measurements at different speeds, an increase in speed leads to a decrease in both downforce and torque, regardless of variations in tool structure; conversely, there is a significant increase in the temperature peaks.

The changes in downforce and torque during friction stir welding are primarily associated with the heat input from the welding. During the welding process, friction occurs between the contact surface of the welding tool and the workpiece being welded, converting the mechanical energy applied to the tool into the heat energy required for welding. According to traditional models of frictional heat generation, an increasing trend in heat generation is observed as rotational speed increases. It is generally believed that adhesion is the main cause of friction between metal materials. For two surfaces in contact to slide relative to each other, a certain amount of shear stress must be applied to one of the surfaces to overcome the intermolecular bonding forces and generate friction. At this point, the frictional shear stress should be equal to the shear stress at which the material yields. According to the Mises yield criterion, the relationship between the normal stress *σ_y_* and shear stress *τ_yie_* when a material yields under pure shear stress state is *τ_yie_ = σ_y_*/(3^1/2^). For the 6××× series aluminum alloy used in this experiment, its yield strength decreases with increasing temperature, leading to a decrease in shear stress as well. Therefore, as rotational speed increases and temperature at the friction interface rises, matrix material yield strength gradually decreases along with the total amount of generated frictional heat resulting in a downward trend in both downforce and torque.

### 3.4. Friction and Wear Characteristics of Bionic Structure Welding Tool

**Figure 8:**
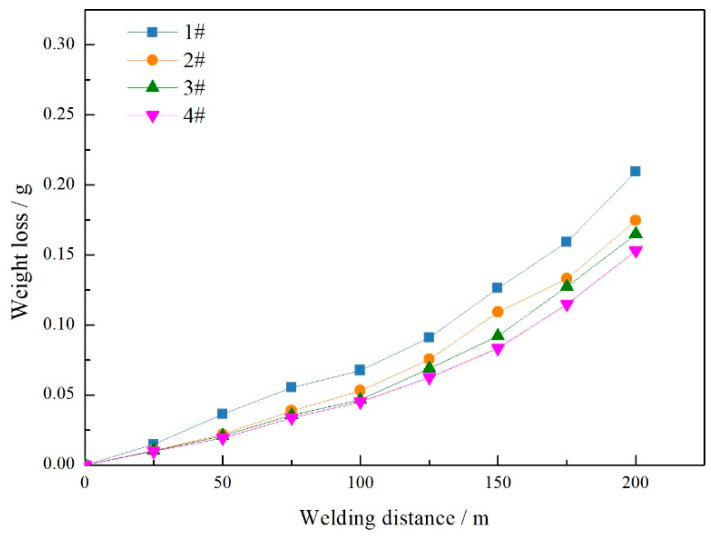
Comparison of weight loss with different tools at welding.

After carrying out the above research, it was found that welding tools with non-smooth structures have the effect of reducing downforce and torque. This indicates a reduction in the coefficient of friction and frictional force, which will affect the wear resistance of the tools during use. To assess the influence of the structural shape of the tool on its service life, a process with a rotational speed of 1500 r/min and a welding speed of 500 mm/min was selected for this study. The method involved simulating actual welding to test all specifications of tools with a length of 200 m. The weight loss of each tool during the welding process was measured every 25 m and the results are shown in Figure 8. The graph shows that all tools show a trend of slow initial weight loss followed by accelerated wear during the welding process. A comparison between the different types of welding tools showed that those without non-smooth structures experienced a significant amount of weight loss, which reached 0.2096 g at a length of 200 m. Conversely, the tools with non-smooth structures showed relatively lower weight losses, with the lowest being recorded for welding tool 4# at 0.1529 g at a length of 200 m, a 27% reduction compared to those without such a structure.

**Figure 9:**
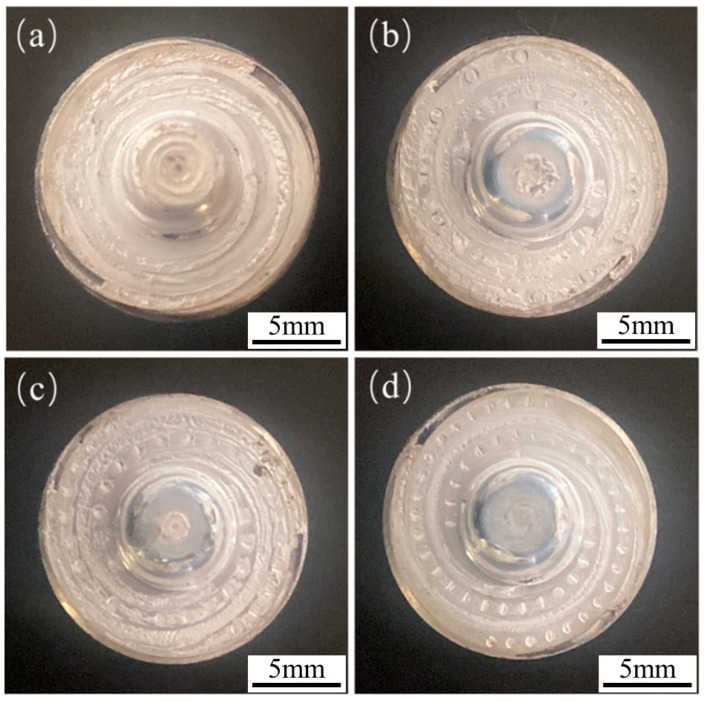
Morphology of the tools after welding: (**a**) 1#, (**b**) 2#, (**c**) 3#, (**d**) 4#.

**Figure 10:**
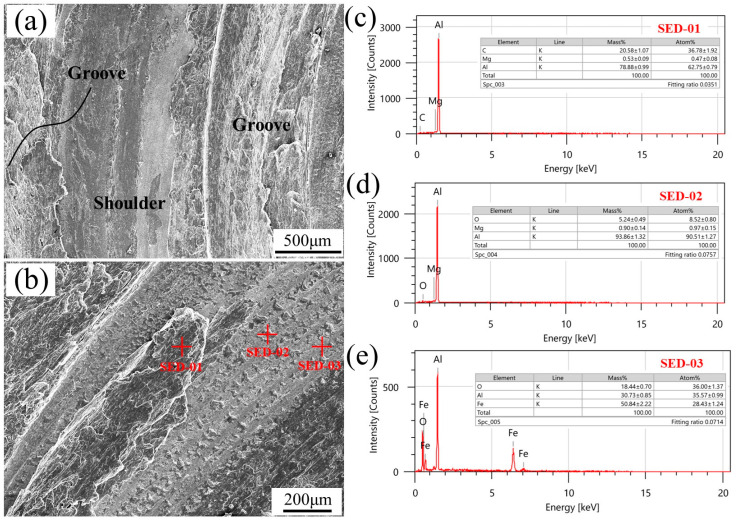
SEM observation and energy spectrum analysis of 1# tool after welding: (**a**,**b**) SEM image, (**c**–**e**) Energy spectrum analysis.

By observing the surface morphology of the newly utilized tools, as depicted in Figure 9, it is evident that the shoulder face of the tool with a bionic non-smooth structure appears to be relatively smooth, devoid of conspicuous wear marks, and exhibits minimal aluminum adhesion. The SEM analysis of the shoulder morphology for tool 1# is presented in Figure 10. Figure 10a illustrates a lower magnification SEM image showing the complete filling of grooves and platforms on the shoulder with aluminum chips post-welding, at an equal height to that of the platform. Additionally, traces of compression friction from aluminum chips are observed on the surface of the shoulder platform. Further magnification (as shown in Figure 10b) reveals large and numerous small particle-like attachments adhering to the platform surface. The results of the energy spectrum analysis conducted on these attachments are displayed in Figure 10c–e, indicating their composition of aluminum alloy particles both large and small in size, signifying a strong adhesion between tool 1#s shoulder platform and aluminum alloy material. Despite careful examination, no discernible signs of friction or wear were found on the substrate of the shoulder platform; this may be attributed to the high hardness and wear resistance properties inherent to raw materials used for tool fabrication or it could be due to the limited duration of the experiment duration without substantial wear.

**Figure 11:**
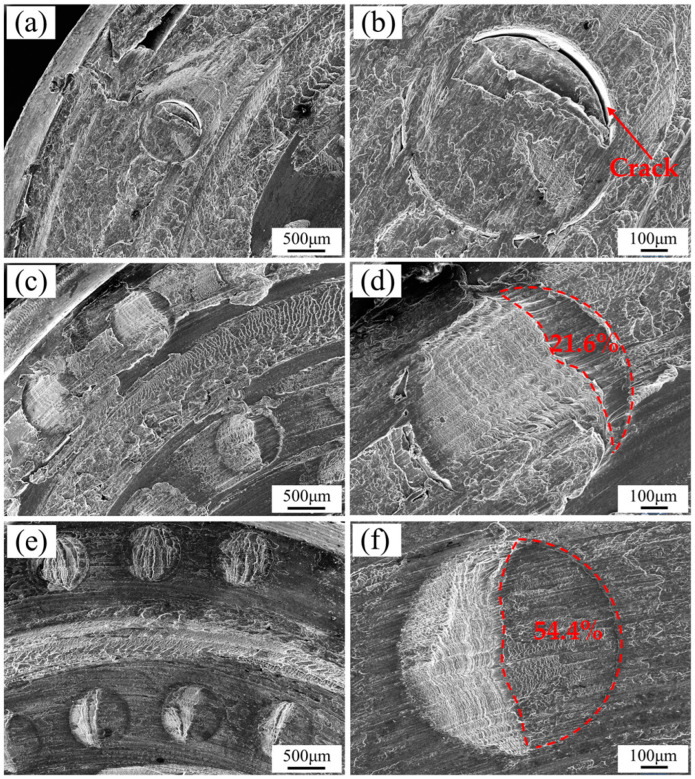
SEM observation of the morphology of the bionic non-smooth structure tools after welding: (**a**,**b**), 2 #; (**c**,**d**), 3 #; (**e**,**f**), 4 #.

The shoulders of the 2#, 3#, and 4# welding tools were observed using SEM after use, and the results are presented in Figure 11. Figure 11a,c,e depict the low magnification morphology of the respective shoulders. The spiral grooves on the shoulders and bionic non-smooth pits between the grooves are clearly visible in these figures. The spiral grooves are filled with aluminum shavings, and the surface exhibits scale-like friction marks. Additionally, some aluminum shavings filled the non-smooth pits on the shoulder’s face, with a small amount also adhering to the platform between the grooves. Under high magnification, the morphologies were observed and are presented in Figure 11b,d,f, respectively. Upon comparison and analysis of these non-smooth pits, it was determined that the filling degree of aluminum chips in the pits varies with changes in pit distribution density. The pits of 2# with the widest spacing were predominantly filled with aluminum chips, and only exhibited a peeling crack with a large arc surface of approximately 120° at the front end with reference to the welding rotation direction. Both 3# and 4#’s pits were partially filled with aluminum chips, showing an unfilled area at their fronts. The unfilled area accounts for about 21.6% of the pit of 3#, while more than 54.4% of the pits of 4# remain unfilled. Simultaneously, it was observed that the adhesion capabilities of aluminum alloy chips on the shoulder platform surface decreased significantly with an increase in pit density. Specifically, a substantial amount of aluminum alloy adhered to the shoulder platform of tool 2#, while no large aluminum alloy attachments were found between the pits and grooves on the shoulder of tool 3#. Nonetheless, some aluminum alloy attachments were still generated through friction and scraping between the pits and grooves. No significant aluminum alloy attachments were present between the pits on the shoulder of tool 4# and between the pits and grooves. This indicates that non-smooth pits can diminish the adhesion force between friction surfaces, and adjusting the pit distribution density can optimize the friction surface adhesion to aluminum alloys, thereby reducing adhesion, drag, and wear.

After analyzing the above experimental results, it was found that the non-smooth pit structure in this study effectively reduces wear and resistance. The main reasons for this are as follows: (1) When concave pits with non-smooth structures come into contact and rub against plastic metal, a certain gap is formed at the contact interface. The phenomenon of contact angle hysteresis, similar to that produced by the non-smooth unit cells in the biomimetic surface, increases the advance angle of the high-temperature plastic metal on the sample surface, causing the plastic metal to form a void gap on the non-smooth surface. (2) The non-smooth pit structure of the shoulder can trap air, forming a solid/gas composite surface, which reduces the inherent contact angle between interfaces and further reduces wetting performance. In addition, air trapped in narrow pits expands due to frictional heat during the welding process, displacing the plastic metal entering the pits and reducing viscosity, drag, and wear. (3) The presence of pits on the contact surface between the shoulder and the base material creates a discontinuity that provides good resistance to adhesive wear. Pits also effectively block adhesive wear at certain points on the shoulder surface.

## 4. Conclusions

(1) When comparing the distribution density of non-smooth pits, it was observed that as the spacing between pits decreases and the number of pits increases, the peak downforce, torque, and temperature during the welding process exhibit a decreasing trend. This indicates that the application of non-smooth pit structures on the shoulder of the welding tool can attenuate downforce, reduce friction, and lower torque. When comparing measured values at different speeds, it was found that at 1500 r/min there are similar changes in downforce, torque, and temperature for different non-smooth structure tools compared to those at 1000 r/min. As speed increases, regardless of what tool structure changes occur, there is a decrease in both downforce and torque while an increase in temperature peaks is observed.
(2) The welding experiment results have shown that the wear weight loss of the non-smooth structure tools is significantly reduced, with the lowest weight loss of tool 4# being only 0.1529 g, which is 27% lower than that of tools without this structure.
(3) Observing the surface morphology after welding, it was found that a large amount of sheet-like and granular aluminum adhesive was adhered to the shoulder of the conventional tool. However, as the density of non-smooth pits increased, the amount of aluminum adhesive on the shoulder of the welding tool gradually decreased. There were no large aluminum chips between the pits or the pits and grooves of the 4# tool. Therefore, welding tools with non-smooth concave surfaces have characteristics such as wear resistance, viscosity reduction, and an improved service life.

## Figures and Tables

**Table 1:** Chemical composition of H13 hot-working die steel (wt%).

C	Si	Mn	Cr	Mo	V	P	S	Fe
0.32–0.45	0.80–1.20	0.20–0.50	4.75–5.50	1.10–1.75	0.80–1.20	≤0.30	≤0.30	Remainder

**Table 2:** Chemical composition of 6061-T6 aluminum alloy (wt%).

Si	Fe	Cu	Mn	Mg	Cr	Zn	Ti	Al
0.4~0.8	≤0.7	0.1~0.4	0.15	0.6~1.2	0.04	0.25	0.15	Remainder

## Data Availability

The data that support the findings of this study are available from the corresponding author upon reasonable request.
